# Supplementary material for: A Likelihood Approach for Real-Time Calibration of Stochastic Compartmental Epidemic Models
Source: PLoS Comput Biol. 2017 Jan 17;13(1):e1005257. doi: 10.1371/journal.pcbi.1005257 (PMC5240920; doi:10.1371/journal.pcbi.1005257)
Supplement: S1 File — (TAR.GZ) [file pcbi.1005257.s014.tar.gz › HSPH_Online-SI-Revision/Additions/EnKF08/n100-extreme-SEITR/n100-extreme-SEITR_EnKF_table-8.pdf]

1	R0	{1.38654, 9.85565}	{2.58996, 6.89062}	{{1.45209, 3.41984}, {3., 9.90024}}	{89.4612, 30.4528}	inf	369.	2130.54	{839.248, 1487.73}	308.933	0.474907	newly	522.	2147.24	{497., 2131.}	313.1	0.399198	Reff	1.36736	0.461245	{0.0255704, 0.61909}	68.5248	ar	92 379.	182 742.	{174 539., 188 154.}	97.8182
2	R0	{2.36629, 12.2029}	{2.18487, 10.0083}	{{0.908027, 1.99107}, {3., 17.1085}}	{30.641, 39.6183}	inf	10.	24 987.2	{8529.58, 53 058.6}	178 380.	3.09926	newly	20.	23 381.1	{6289., 39 718.}	116 806.	2.85749	Reff	2.3656	1.29499	{0.358629, 2.22214}	45.6151	ar	99 022.	124 396.	{65 311., 173 397.}	39.0706
3	R0	{2.32253, 12.836}	{2.12825, 8.90294}	{{0.315493, 2.00079}, {3., 16.4646}}	{33.3528, 42.9011}	inf	18.	9980.71	{2521.96, 22 477.4}	36 865.6	2.4104	newly	26.	21 326.7	{2644., 69 369.}	81 925.6	2.58239	Reff	2.32139	1.81346	{0.247526, 2.92998}	34.5115	ar	98 997.	84 429.9	{18 887., 109 283.}	27.0702
4	R0	{1.79075, 11.2058}	{2.28422, 10.0375}	{{1.46424, 1.99893}, {3., 17.0679}}	{40.5183, 40.9864}	inf	68.	25 724.1	{5046.15, 55 949.4}	26 419.7	2.266	newly	88.	25 025.2	{2559., 61 989.}	28 337.8	2.24874	Reff	1.78676	1.29839	{0.284967, 2.22035}	37.4502	ar	96 920.	131 308.	{76 356., 187 460.}	43.4955
5	R0	{2.26952, 9.62371}	{1.75739, 7.77152}	{{0.316004, 1.54538}, {3., 14.605}}	{43.4364, 48.5514}	inf	2.	8677.12	{1751.28, 19 350.5}	173 442.	3.04545	newly	5.	22 808.2	{1216., 81 381.}	456 064.	3.23731	Reff	2.26934	1.54562	{0.220917, 2.8806}	44.385	ar	98 881.	74 263.7	{13 854., 108 766.}	38.4741
6	R0	{1.57191, 6.89004}	{2.25107, 9.3752}	{{0.682205, 2.10869}, {3., 14.0967}}	{63.8122, 61.9173}	inf	11.	17 276.9	{5029.83, 39 059.9}	143 874.	3.0223	newly	22.	21 266.8	{4834., 52 689.}	96 567.	2.79301	Reff	1.57147	1.68281	{0.319912, 2.34073}	37.9137	ar	95 046.	128 214.	{54 603., 211 728.}	51.3075
7	R0	{1.99226, 17.2339}	{2.27492, 6.47265}	{{1.46732, 3.41084}, {3., 9.94036}}	{35.2116, 62.4423}	inf	713.	4057.14	{796.904, 9773.3}	295.682	0.319252	newly	648.	5930.95	{774., 27 593.}	821.276	0.502357	Reff	1.94486	0.580311	{0.0970854, 1.46786}	71.3304	ar	97 992.	191 088.	{172 577., 253 210.}	95.0041
8	R0	{2.63369, 15.1879}	{2.29912, 10.6301}	{{1.31578, 7.63301}, {3., 17.1068}}	{26.7727, 38.0964}	inf	35.	25 378.6	{3334.01, 49 808.2}	48 704.9	2.52745	newly	41.	21 162.9	{3089., 31 142.}	51 516.8	2.49523	Reff	2.6309	1.19915	{0.309617, 1.85881}	54.8639	ar	99 451.	125 970.	{84 000., 169 885.}	34.2201
9	R0	{2.53271, 9.90552}	{2.06229, 7.91294}	{{0.316327, 2.00079}, {3., 17.1792}}	{34.0091, 57.1716}	inf	2.	9842.12	{2192.02, 24 844.5}	492 006.	3.53886	newly	8.	28 955.3	{2335., 79 782.}	361 842.	3.20114	Reff	2.53258	1.79373	{0.25347, 2.95608}	36.1056	ar	99 355.	82 049.5	{16 489., 107 681.}	29.8728
10	R0	{2.48791, 18.9003}	{2.60008, 6.7244}	{{2.15033, 4.21854}, {3., 9.90209}}	{18.133, 64.4217}	inf	205.	21 867.	{6243.55, 57 249.3}	6931.21	1.68011	newly	173.	23 033.4	{4224., 62 597.}	13 214.1	1.92377	Reff	2.47216	0.887516	{0.319969, 2.08233}	64.0996	ar	99 291.	144 561.	{70 648., 236 647.}	57.017
11	R0	{2.84414, 18.4765}	{2.42997, 5.45971}	{{1.7618, 1.07453}, {3., 13.0891}}	{20.2614, 70.4505}	inf	68.	19 418.3	{4430.65, 53 188.9}	18 754.3	2.11517	newly	76.	22 679.5	{2648., 64 947.}	29 742.8	2.24874	Reff	2.83774	0.593708	{0.207655, 1.30345}	79.0782	ar	99 654.	140 665.	{71 906., 242 528.}	55.8676
12	R0	{2.29369, 14.622}	{2.57829, 6.68378}	{{2.16991, 4.24266}, {3., 9.77022}}	{20.2579, 54.4819}	inf	100.	22 052.5	{7474.97, 34 840.2}	15 540.1	2.07497	newly	114.	25 675.2	{5645., 72 340.}	22 422.1	2.17749	Reff	2.28683	0.964827	{0.297331, 2.03937}	57.8692	ar	98 982.	154 077.	{106 944., 223 429.}	60.1989
13	R0	{2.73413, 16.7529}	{2.22365, 5.44297}	{{0.839497, 2.17325}, {3., 10.0977}}	{27.6167, 67.5103}	inf	62.	32 931.3	{8888.59, 71 872.3}	42 119.6	2.49333	newly	67.	42 756.9	{13 417., 85 646.}	63 716.3	2.66927	Reff	2.72932	1.14269	{0.404421, 1.74281}	58.1327	ar	99 543.	145 866.	{77 799., 196 909.}	55.3554
14	R0	{1.79554, 16.5943}	{1.97215, 8.47231}	{{0.391901, 2.89217}, {3., 13.1185}}	{59.8345, 48.9444}	inf	652.	5055.02	{2550.11, 7563.03}	429.876	0.532247	newly	541.	4249.58	{1065., 9664.}	685.505	0.63842	Reff	1.75512	0.360674	{0.0615441, 0.632144}	79.9348	ar	96 821.	188 699.	{179 306., 213 732.}	94.8951
15	R0	{1.91429, 13.7771}	{2.40865, 6.86625}	{{1.48432, 3.43108}, {3., 9.88597}}	{38.3866, 50.1618}	inf	266.	3055.07	{1202.15, 2762.17}	718.224	0.691344	newly	278.	3356.38	{708., 3868.}	1119.59	0.780182	Reff	1.89691	0.601138	{0.22104, 1.03684}	70.1497	ar	97 541.	184 969.	{171 903., 198 633.}	89.6321
16	R0	{1.42094, 10.5871}	{2.0135, 4.21863}	{{1.04831, 2.64544}, {3., 5.00848}}	{61.8432, 60.1531}	inf	260.	15 866.5	{8730.21, 38 762.8}	4075.41	1.49635	newly	379.	17 763.4	{6706., 44 847.}	4586.92	1.50973	Reff	1.40736	0.371331	{0.0793165, 0.816679}	74.3145	ar	93 044.	157 254.	{86 983., 217 460.}	75.5189
17	R0	{1.45515, 6.16466}	{1.97878, 9.17371}	{{0.283608, 2.00079}, {3., 16.6874}}	{62.7337, 93.4972}	inf	5.	10 550.9	{3049.21, 22 982.4}	351 598.	3.38439	newly	11.	22 853.6	{2077., 72 446.}	207 660.	2.97548	Reff	1.45502	1.70356	{0.248104, 2.90498}	54.7317	ar	93 254.	85 295.9	{16 491., 108 389.}	28.5758
18	R0	{2.85669, 13.2416}	{2.44158, 8.95998}	{{1.88247, 2.11086}, {3., 12.9023}}	{19.2785, 36.0879}	inf	3.	19 734.8	{6608.36, 38 599.5}	179 308.	3.1284	newly	8.	27 887.1	{4475., 74 209.}	348 489.	3.32664	Reff	2.85618	1.84121	{0.618917, 2.79639}	36.5426	ar	99 644.	136 348.	{85 021., 199 681.}	43.5019
19	R0	{2.56417, 17.9558}	{2.07459, 4.58153}	{{1.05525, 2.6454}, {3., 4.98953}}	{34.9173, 74.579}	inf	91.	17 123.7	{7736.48, 58 343.7}	10 947.5	1.87035	newly	91.	16 868.1	{6450., 45 033.}	18 436.3	2.09654	Reff	2.55604	0.362306	{0.0734588, 0.738605}	85.8255	ar	99 389.	169 511.	{99 778., 270 207.}	73.0357
20	R0	{2.27517, 11.1864}	{2.27482, 8.61321}	{{1.11008, 2.10871}, {3., 12.8989}}	{30.6486, 38.2831}	inf	8.	17 340.5	{5057.51, 40 913.7}	108 278.	2.90213	newly	17.	27 532.	{4410., 83 925.}	161 853.	2.96209	Reff	2.27446	1.67745	{0.500153, 2.70142}	33.4847	ar	98 894.	132 048.	{58 940., 199 496.}	46.5115
21	R0	{2.35735, 10.4735}	{1.8668, 8.35847}	{{0.402435, 1.44399}, {3., 16.2996}}	{38.3542, 45.3342}	inf	5.	8409.55	{1998.7, 19 800.5}	168 091.	3.04505	newly	5.	22 572.6	{1337., 75 908.}	451 352.	3.23371	Reff	2.35707	1.65338	{0.287485, 2.93827}	40.0517	ar	99 036.	76 496.3	{18 190., 108 915.}	32.1826
22	R0	{1.79551, 11.9179}	{2.52025, 7.3427}	{{2.16555, 4.21574}, {3., 14.7337}}	{44.9945, 43.6092}	inf	143.	20 859.5	{3798.67, 39 797.3}	9254.04	1.77074	newly	205.	22 802.1	{3743., 58 138.}	11 023.	1.79628	Reff	1.78616	1.00315	{0.394291, 2.18142}	52.0883	ar	97 033.	149 968.	{88 463., 234 029.}	61.4856
23	R0	{1.23545, 6.31687}	{2.18945, 6.24565}	{{0.840216, 2.17328}, {3., 10.0964}}	{95.3277, 45.2846}	inf	66.	24 368.8	{4003.49, 40 971.9}	19 712.	2.14625	newly	213.	34 767.9	{1852., 80 804.}	16 224.	1.97749	Reff	1.23161	1.07174	{0.275162, 2.16845}	38.67	ar	88 739.	136 234.	{80 889., 200 517.}	58.4399
24	R0	{2.24089, 10.1789}	{2.32925, 8.75319}	{{1.03228, 2.10877}, {3., 12.8984}}	{31.0637, 35.4342}	inf	4.	17 498.9	{5164.24, 37 138.8}	291 549.	3.35644	newly	6.	25 945.6	{4318., 85 113.}	432 326.	3.43435	Reff	2.24062	1.70107	{0.48171, 2.85611}	31.8818	ar	98 868.	130 817.	{65 489., 196 876.}	46.3861
25	R0	{1.57298, 10.2607}	{2.40007, 5.13154}	{{1.7628, 1.06476}, {3., 13.073}}	{52.581, 65.3131}	inf	107.	25 419.6	{4856.69, 54 176.4}	17 802.3	2.06373	newly	146.	28 743.4	{4877., 66 974.}	19 588.	2.07929	Reff	1.56721	0.665455	{0.310045, 1.35356}	59.0831	ar	94 957.	170 400.	{116 814., 250 249.}	80.5515
26	R0	{1.2154, 8.2576}	{1.88588, 3.89656}	{{0.0654495, 0.928741}, {3., 5.69817}}	{115.652, 54.3715}	inf	374.	5825.29	{289.257, 20 422.8}	1161.63	0.841325	newly	559.	6451.12	{290., 18 492.}	1070.82	0.812394	Reff	1.19834	0.319438	{0.00446002, 0.446158}	77.3636	ar	88 421.	178 110.	{160 295., 236 154.}	103.67
27	R0	{2.22841, 18.7983}	{2.13973, 4.44078}	{{0.0901357, 0.925612}, {3., 5.73683}}	{42.067, 76.3767}	inf	579.	4952.01	{462.148, 12 468.3}	496.96	0.610236	newly	487.	5063.23	{423., 15 265.}	946.758	0.793458	Reff	2.1875	0.303723	{0.0136502, 0.466249}	86.1155	ar	98 756.	146 621.	{102 251., 181 980.}	55.2574
28	R0	{1.27778, 5.36767}	{1.84242, 8.6547}	{{0.449598, 2.00079}, {3., 15.0036}}	{75.7624, 100.109}	inf	15.	8816.65	{3228.33, 17 018.}	48 881.4	2.53744	newly	37.	18 891.6	{2502., 70 051.}	50 958.9	2.35046	Reff	1.27717	1.58953	{0.176233, 2.95444}	68.0422	ar	89 843.	75 950.3	{21 813., 109 539.}	31.7629
29	R0	{2.38042, 15.8901}	{2.09924, 5.47918}	{{0.839669, 2.17318}, {3., 10.0792}}	{33.1553, 65.6833}	inf	75.	31 381.	{10 209.8, 62 054.3}	24 805.5	2.30067	newly	93.	43 289.	{15 175., 100 198.}	46 447.3	2.53379	Reff	2.37421	1.1388	{0.406734, 1.97441}	52.7913	ar	99 112.	145 632.	{84 779., 204 123.}	55.1428
30	R0	{2.53188, 12.7607}	{1.95673, 8.66755}	{{0.574232, 2.00079}, {3., 16.7292}}	{33.6477, 43.4081}	inf	1.	9980.54	{3007.03, 22 012.8}	199 511.	3.17622	newly	3.	21 204.1	{2616., 64 491.}	706 705.	3.5407	Reff	2.5317	1.69849	{0.570869, 2.73582}	37.1528	ar	99 317.	83 341.5	{21 425., 109 321.}	31.8679
31	R0	{1.24482, 10.5471}	{2.10821, 8.22709}	{{0.411382, 2.9315}, {3., 12.94}}	{105.454, 41.8143}	inf	904.	4593.42	{3077.24, 3077.24}	261.968	0.446824	newly	1087.	3735.74	{1324., 4142.}	246.033	0.374305	Reff	1.19689	0.33912	{0.0712466, 0.500499}	71.6666	ar	89 097.	186 291.	{179 443., 187 133.}	109.088
32	R0	{2.30446, 17.9607}	{2.54528, 7.39111}	{{2.15968, 4.18861}, {3., 14.525}}	{17.3298, 58.8484}	inf	288.	23 970.	{7478.95, 54 958.1}	5515.03	1.61084	newly	254.	24 111.7	{4995., 60 684.}	9394.57	1.8032	Reff	2.28427	0.892052	{0.352537, 2.05323}	60.9481	ar	99 028.	148 788.	{96 670., 215 611.}	55.5763
33	R0	{2.31579, 11.8236}	{1.86988, 7.29847}	{{0.452369, 2.00079}, {3., 15.3617}}	{39.4219, 50.7013}	inf	22.	7366.72	{617.469, 14 555.5}	26 209.7	2.19328	newly	33.	22 477.2	{1119., 83 012.}	68 013.5	2.40843	Reff	2.31442	1.71378	{0.450508, 2.99997}	39.1282	ar	99 012.	77 154.	{16 737., 108 908.}	37.0629
34	R0	{1.6678, 10.6744}	{2.447, 7.29249}	{{2.13696, 4.20361}, {3., 14.7358}}	{54.1445, 44.3283}	inf	174.	23 150.8	{5463.23, 48 748.5}	9470.63	1.80221	newly	267.	25 158.8	{3677., 58 389.}	9325.84	1.74359	Reff	1.65861	0.997133	{0.230343, 2.18362}	52.2186	ar	95 809.	164 750.	{101 522., 231 993.}	74.521
35	R0	{1.65476, 7.07559}	{1.85556, 9.05447}	{{0.483301, 2.00079}, {3., 16.2753}}	{56.1612, 75.9753}	inf	1.	9727.26	{2581.42, 21 573.7}	121 491.	2.91698	newly	10.	19 446.6	{1826., 70 402.}	194 366.	2.94051	Reff	1.65458	1.60427	{0.405144, 2.86373}	48.9608	ar	95 746.	75 958.8	{15 377., 109 540.}	37.567
36	R0	{2.71807, 18.5959}	{2.58594, 7.28817}	{{2.1611, 4.24575}, {3., 9.88824}}	{15.4412, 60.8077}	inf	74.	21 063.3	{6690.95, 34 056.4}	23 566.6	2.2811	newly	61.	22 369.5	{5880., 53 575.}	36 571.3	2.42096	Reff	2.71258	0.936428	{0.41627, 2.11131}	65.4783	ar	99 540.	150 074.	{98 092., 217 812.}	55.8658
37	R0	{2.6202, 12.7552}	{2.12871, 5.43324}	{{0.839569, 2.17328}, {3., 10.08}}	{31.029, 58.4745}	inf	2.	29 373.6	{10 048.5, 55 027.9}	419 522.	3.52258	newly	7.	43 944.6	{11 639., 98 120.}	627 680.	3.63821	Reff	2.61991	1.15773	{0.455437, 2.1737}	56.3906	ar	99 426.	139 653.	{91 915., 201 739.}	47.8001
38	R0	{1.47672, 9.81651}	{2.17779, 4.39547}	{{1.14427, 2.65541}, {3., 5.00825}}	{63.755, 56.5356}	inf	196.	14 049.2	{3502.14, 26 094.6}	4131.68	1.45276	newly	329.	15 816.6	{3151., 46 096.}	4707.49	1.47282	Reff	1.46491	0.394566	{0.0508073, 0.82575}	73.5602	ar	93 815.	155 111.	{90 404., 219 904.}	70.3275
39	R0	{1.90004, 12.3313}	{2.50324, 6.56037}	{{2.15887, 4.26029}, {3., 9.79206}}	{38.1409, 50.7016}	inf	121.	21 535.8	{5284.26, 33 466.3}	12 420.8	1.96835	newly	167.	25 014.5	{5298., 59 170.}	14 878.8	1.99601	Reff	1.89271	0.941102	{0.31603, 2.18549}	55.1807	ar	97 413.	153 939.	{83 421., 225 102.}	67.0431
40	R0	{2.64218, 18.5237}	{2.24849, 6.38789}	{{0.843017, 2.17565}, {3., 10.0975}}	{27.7134, 65.8713}	inf	208.	27 641.1	{10 583.3, 49 357.9}	9807.21	1.92377	newly	187.	33 989.6	{8597., 69 680.}	18 076.3	2.13353	Reff	2.62693	1.05533	{0.403563, 1.73408}	59.8266	ar	99 474.	136 129.	{73 551., 193 694.}	47.666
41	R0	{1.6945, 7.17996}	{1.92665, 7.47096}	{{0.506202, 2.00079}, {3., 15.3078}}	{51.6366, 69.9846}	inf	3.	8442.26	{2913.96, 18 334.5}	168 745.	3.09123	newly	9.	24 981.5	{2772., 72 396.}	277 472.	3.10299	Reff	1.69433	1.70784	{0.486103, 2.97494}	46.3426	ar	96 332.	77 870.9	{16 345., 109 224.}	33.6281
42	R0	{1.84559, 18.0074}	{2.50028, 7.25096}	{{1.46548, 3.39329}, {3., 9.89222}}	{44.9507, 59.7334}	inf	1046.	2509.54	{964.953, 1476.25}	84.0138	0.144063	newly	870.	2708.3	{583., 2086.}	228.894	0.280907	Reff	1.78061	0.491831	{0.0525665, 1.10603}	73.6851	ar	97 358.	182 566.	{173 715., 207 239.}	87.5205
43	R0	{2.24965, 17.0361}	{2.50579, 7.87884}	{{2.15923, 4.27174}, {3., 14.7195}}	{20.6545, 53.8356}	inf	172.	20 382.	{5993.03, 31 532.6}	8323.18	1.79319	newly	161.	21 437.	{4167., 51 858.}	13 216.	1.94291	Reff	2.23721	1.01982	{0.26055, 2.18561}	54.74	ar	98 770.	156 451.	{101 953., 228 113.}	63.6265
44	R0	{2.54324, 15.7967}	{2.60676, 7.53798}	{{2.16094, 4.24602}, {3., 14.7366}}	{15.3528, 52.6286}	inf	68.	19 950.1	{4935.11, 32 856.4}	16 951.3	2.09758	newly	107.	19 850.2	{5748., 37 757.}	18 451.6	2.10462	Reff	2.53714	0.977612	{0.332056, 2.17714}	61.4679	ar	99 354.	154 395.	{108 786., 216 045.}	60.4258
45	R0	{2.54943, 14.8032}	{2.30377, 9.82257}	{{1.582, 1.99079}, {3., 17.0591}}	{25.6101, 41.9871}	inf	40.	25 662.7	{7713.03, 55 925.6}	44 923.6	2.49932	newly	50.	23 748.4	{6627., 45 042.}	47 398.4	2.51459	Reff	2.54637	1.36021	{0.443804, 2.22209}	47.7544	ar	99 380.	131 984.	{78 648., 173 545.}	40.7494
46	R0	{2.37755, 12.6461}	{1.83053, 8.18183}	{{0.450814, 2.00079}, {3., 16.4725}}	{39.1775, 47.4773}	inf	8.	10 133.9	{2156.85, 25 240.4}	63 237.1	2.60151	newly	26.	22 668.9	{1929., 72 369.}	87 088.7	2.57631	Reff	2.37696	1.51388	{0.367385, 2.82315}	43.7257	ar	99 096.	75 839.1	{16 377., 106 494.}	37.1739
47	R0	{1.69279, 11.6636}	{2.52968, 4.74638}	{{1.74588, 1.13152}, {3., 12.8574}}	{49.4388, 63.7983}	inf	138.	21 397.8	{4632.63, 49 032.6}	10 493.2	1.85958	newly	167.	23 545.2	{3806., 43 323.}	13 998.9	1.96149	Reff	1.68417	0.535702	{0.0895933, 0.89509}	68.9392	ar	96 214.	151 471.	{91 547., 239 782.}	62.6395
48	R0	{1.82402, 10.0038}	{2.28709, 9.90689}	{{1.45282, 4.38646}, {3., 17.1032}}	{43.1328, 36.8583}	inf	25.	29 422.	{6105.41, 74 181.4}	89 057.7	2.77744	newly	39.	27 219.9	{6520., 80 576.}	69 694.7	2.61927	Reff	1.82252	1.34495	{0.293037, 2.22219}	36.9912	ar	97 076.	137 962.	{78 856., 194 176.}	50.6301
49	R0	{1.6424, 11.1667}	{2.45527, 6.48111}	{{2.16233, 4.12986}, {3., 14.7923}}	{55.9611, 50.7178}	inf	267.	23 049.7	{4427.55, 55 747.3}	5781.46	1.61588	newly	380.	28 908.6	{5046., 72 944.}	7508.47	1.67272	Reff	1.62686	1.04425	{0.228321, 2.18348}	53.2538	ar	95 546.	162 212.	{83 962., 238 917.}	77.3147
50	R0	{2.05638, 14.3048}	{2.11541, 5.88279}	{{0.84223, 2.16804}, {3., 8.36288}}	{42.5989, 60.0775}	inf	167.	25 163.6	{5856.19, 40 410.3}	11 288.1	1.92346	newly	176.	35 282.4	{5179., 85 126.}	19 948.9	2.08536	Reff	2.0461	1.06838	{0.406137, 1.86905}	51.689	ar	98 194.	131 556.	{73 437., 198 702.}	42.9508
